# Impact of Heat Stress on Expression of Wheat Genes Responsive to Hessian Fly Infestation

**DOI:** 10.3390/plants11111402

**Published:** 2022-05-25

**Authors:** Jiazheng Yuan, Jordan O’Neal, Daria Brown, Lieceng Zhu

**Affiliations:** Plant Interaction Laboratory, Department of Biological and Forensic Sciences, Fayetteville State University, Fayetteville, NC 28301, USA; jyuan@uncfsu.edu (J.Y.); joneal@broncos.uncfsu.edu (J.O.); dbrown69@broncos.uncfsu.edu (D.B.)

**Keywords:** heat stress, wheat resistance, Hessian fly, RNA sequencing, transcript expression, candidate genes

## Abstract

Heat stress compromises wheat (*Triticum aestivium*) resistance to Hessian fly (HF, *Mayetiola destructor* (Say)). This study aimed to investigate the impact of heat stress on transcript expression of wheat genes associated with resistance to HF infestation under normal and heat-stressed conditions. To this end, ‘Molly’, a wheat cultivar containing the resistance gene *H13*, was subjected to HF infestation, heat stress, and the combination of HF infestation and heat stress. Our RNA-Seq approach identified 21 wheat genes regulated by HF infestation under normal temperatures (18 °C) and 155 genes regulated by HF infestation when plants were exposed to 35 °C for 6 h. Three differentially expressed genes (DEGs) from the RNA-Seq analysis were selected to validate the gene function of these DEGs using the RT-qPCR approach, indicating that these DEGs may differentially contribute to the expression of wheat resistance during the early stage of wheat–HF interaction under various stresses. Moreover, the jasmonate ZIM domain (JAZ) gene was also significantly upregulated under these treatments. Our results suggest that the genes in heat-stressed wheat plants are more responsive to HF infestation than those in plants growing under normal temperature conditions, and these genes in HF-infested wheat plants are more responsive to heat stress than those in plants without infestation.

## 1. Introduction

Hessian fly (HF, *Mayetiola destructor* (Say)) is a destructive pest of wheat (*Triticum aestivum*) in North America and North Africa [1]. Like many plants and their parasites, wheat and HF interact in a gene-for-gene fashion [2]. A single wheat plant can be either resistant or susceptible depending on the virulence or avirulence of the infesting HF biotype(s). When avirulent HF larvae attack a wheat plant containing an effective resistance gene, the plant will exhibit resistance, kill the invading larvae, and develop normally after some initial growth deficits [3]. In contrast, when virulent HF larvae attack a wheat plant with no effective resistance gene(s), nutritive tissues will be induced in the leaf sheath where the HF larval feed in plants known as feeding sites. As a result, the plant will be stunted and eventually killed [4]. Host plant resistance and resistance genes have been exploited in wheat crop production to combat HF and reduce the damage [5,6]. To date, at least 36 HF resistance genes have been identified in wheat and its relative species [7,8,9]. Nevertheless, higher temperatures, even for a short period of time, reduce wheat’s resistance efficiency against HF [10,11,12,13,14]. The shift in the wheat phenotype from resistance to susceptibility due to heat stress is drastic. Moreover, 24 h of heat stress at 35 °C can make 100% of the resistant wheat cultivar ‘Molly’ susceptible to HF infestation [12,15]. 

We have focused on studying the molecular basis of heat-induced loss of host plant resistance to insects using the wheat–HF interaction as a model [14,15,16,17,18]. The molecular and biochemical basis of wheat resistance and susceptibility to HF has been extensively investigated using genomics, functional genomics, and metabolomics technologies. Molecular mapping has located most, if not all, genes in their respective chromosome locations and identified the molecular markers linked to each gene [9,19,20,21,22,23,24,25], which led to the success of map-based cloning of a HF resistance gene, *H13*, in the extensively studied resistant wheat cultivar ‘Molly’ [26]. Functional genomics approaches, such as microarray and RT-qPCR analysis, have revealed numerous genes differentially regulated by HF infestation in resistant and susceptible plants [27,28,29,30,31]. Metabolomics and biochemical analyses have linked the differences in the accumulation of primary and secondary metabolites such as lipids, amino acids, carbohydrates, phenylpropanoid, flavonoids, and phytohormones to the expression of wheat resistance and susceptibility [30,31,32,33,34]. Together, the findings from previous studies have suggested that the resistance of wheat to HF infestation in the incompatible interaction relies on rapid mobilization of plants’ chemical and energy resources to provide the substances and energy needed for synthesizing defense-related compounds. An important step in mobilizing resources is the rapid degradation of primary metabolites (lipid, sugar, proteins, amino acids, etc.) to produce substrates and precursors for synthesizing defensive compounds, such as phenylpropanoids, flavonoids, wax, lectin, insecticidal protein, etc., for cell wall fortification or as antifeedants/toxins to the insect [29,30,31]. In susceptible plants, however, the expression levels of genes pertaining to resource mobilization and biosynthesis of defensive secondary metabolites were reduced, especially during the early stage of wheat–HF interaction [31], suggesting delayed and/or reduced resource mobilization. Phytohormone accumulation is also distinctively different between resistant and susceptible wheat plants. 12-oxo-phytodienoic acid (OPDA) and salicylic acid were highly accumulated in HF feeding tissues of resistant wheat plants during the incompatible interaction, but auxin was highly accumulated in susceptible wheat plants during the compatible interaction [34]. While extensive studies have been conducted to disclose the molecular responses associated with wheat resistance and susceptibility to HF infestation [27,28,33,35], the information on how heat stress, at the molecular level, causes the loss of wheat resistance to HF is limited. In the current research, we treated plants with HF infestation, heat stress, and the combination of heat stress and HF infestation. We sampled the typical HF feeding tissue in wheat plants at 6 h after the initiation of HF attack on plants and/or heat stress for RNA-seq analysis. Via bioinformatical analysis, we identified differentially expressed genes (DEGs) in wheat plants responding to HF infestation under normal and heat conditions and analyzed the impact of heat stress on those genes. The objectives of our study were (1) to understand the impact of heat stress at HF feeding sites in wheat plants, and (2) to identify genes that may be responsible for wheat resistance and heat-induced loss of resistance to HF infestation during the early stage of HF–wheat interaction.

## 2. Results

### 2.1. Differentially Expressed Genes (DEGs) Due to Heat Stress and HF Infestation

The numbers of DEGs generated from differential gene expression analysis are shown in Table 1. Six hours of heat stress at 35 °C, alone or in combination with HF infestation, significantly affected the transcript expression of over 4000 genes, while HF infestation under the control temperature only significantly affected the transcript expression of 21 genes (Table 1). The small number of DEGs caused by HF infestation was likely due to the small number of HF larvae feeding on the wheat plants at the time of sampling. According to the number of DEGs, the effect of the combination of HF infestation and heat stress on the transcript expression of wheat plants was considerably greater than that of heat stress in the uninfested plants but less than that of heat stress in the HF-infested plants. Such results suggested that wheat genes were more responsive to heat stress in HF-infested plants than those in the uninfested plants. Additionally, our results indicated that heat stress, either alone or in combination with HF infestation, downregulated most DEGs (Table 1). In contrast, HF infestation, either under normal or heat-stressed conditions, upregulated most DEGs. The number of genes affected by HF under heat stress was seven times the number of genes regulated by HF infestation under control temperature, suggesting that wheat genes were more responsive to HF infestation under heat stress.

### 2.2. Verification of the DEGs in Response to Heat, HF, and Heat Plus HF Treatments

**Figure 1:**
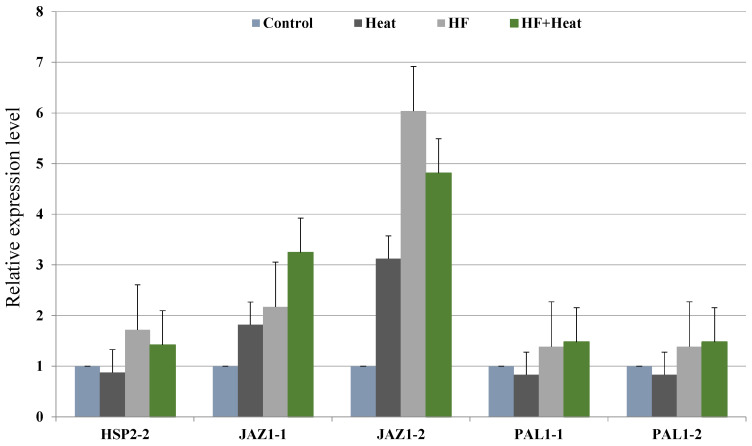
Relative expression level of 3 DEGs in response to heat, HF, and heat plus HF stresses. Error bars were calculated using standard errors of three biological replicates.

To explore the possible function of the wheat DEGs under heat treatment (35 °C for 6 h), HF infestation at the normal temperature (18 °C), and heat plus HF infestation, respectively, six primer pairs of three DEGs from the HF infestation treatment at the normal temperature were selected for RT-qPCR analysis under these stresses to validate the RNA-Seq results (Table 2). Compared with the control, the expression levels of three DEGs (*HSP2-2*, *JAZ1-1*, *JAZ1-2*, *PAL1-1*, *PAL1.2*) were upregulated under the HF and heat plus HF treatments (Figure 1); however, except the *JAZ1* gene, the expression levels of *HSP2* and *PAL1* were downregulated under the heat treatment alone, which displayed a similar trend to the RNA-Seq results, where HF infestation and the combination with HF infestation upregulated the expression of these two genes (Table S6). The *JAZ1* gene was significantly upregulated in response to all three stresses based on RT-qPCR (Figure 1). The heat treatment increased *JAZ* gene expression while it suppressed *HSP2* and *PAL1* gene expression based on the results of the RT-qPCR reaction, which still agrees with what we observed in the RNA-Seq analysis (Table S6), suggesting that heat stress, either alone or in combination with HF infestation, downregulated most DEGs (Table 1). However, one of the HSP2 primer pairs failed to generate a viable result and the DEG of the *JAZ1* gene was only obtained from the HF vs. CK comparison in the RNA-Seq analysis. 

### 2.3. Functional Annotation of DEGs Due to Heat Stress and the Combination of Heat Stress and HF Infestation

**Figure 2:**
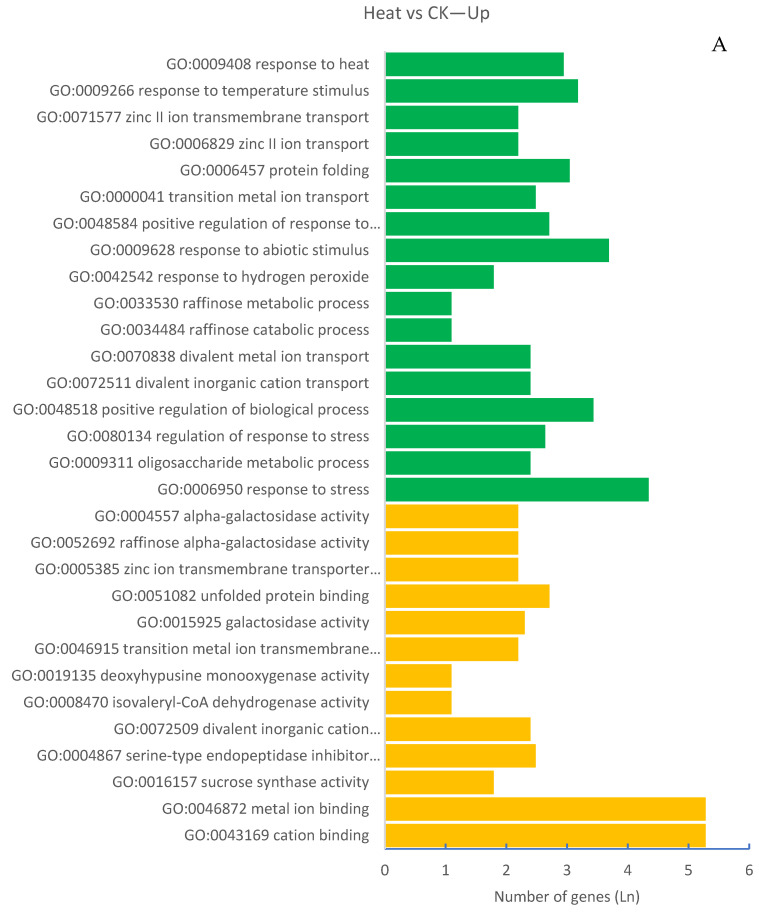

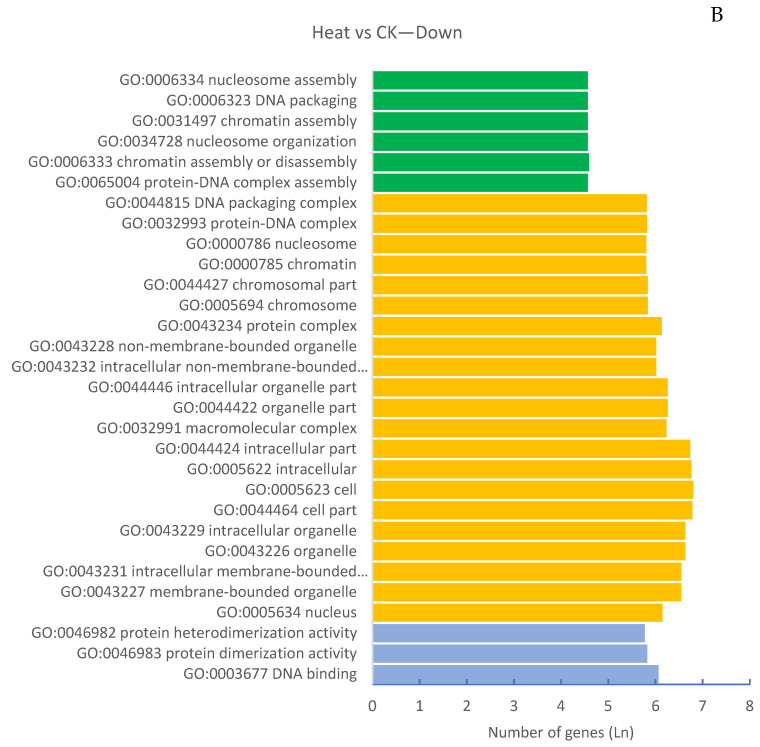

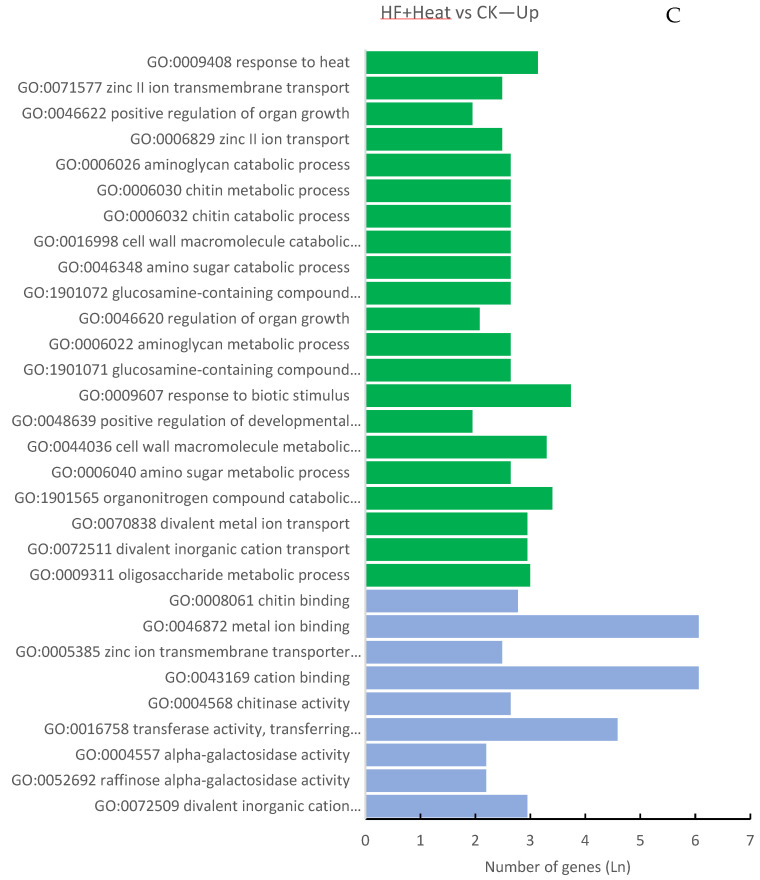

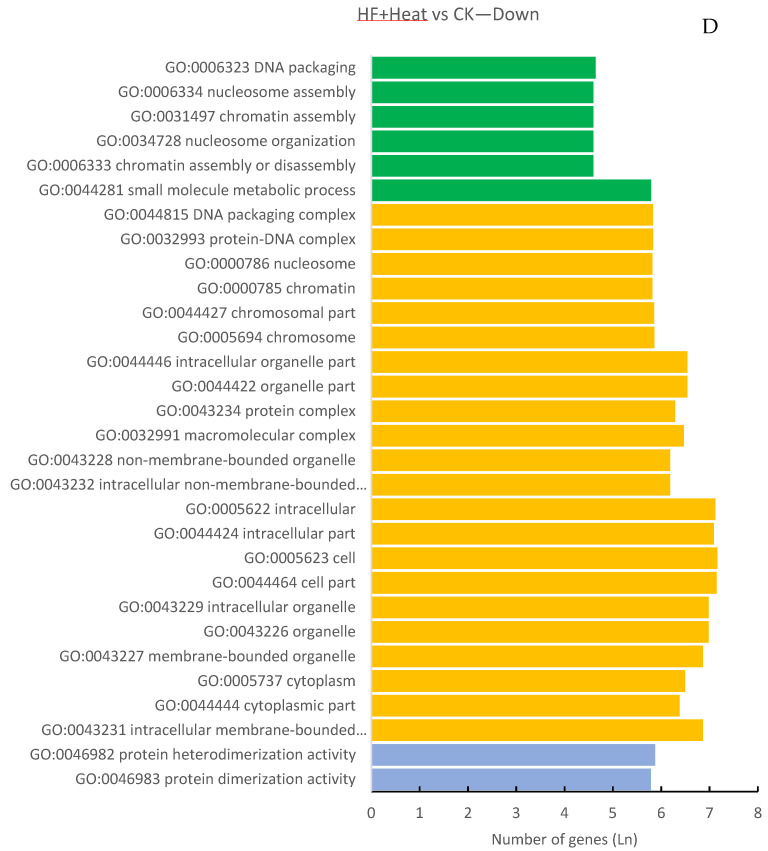
Top 30 GO terms up- or down-enriched by heat stress and the combination of heat stress and HF infestation. *X*-axis: Natural logarithm of the number of DEGs. *Y*-axis: GO number and term. (**A**): Up-enriched by heat stress. (**B**): Down-enriched by heat stress. (**C**): Up-enriched by the combination of heat stress and HF infestation. (**D**): Down-enriched by the combination of heat stress and HF infestation. Green color: Biological process; Orange color: Cellular component; Grey color: Molecular function.

GO enrichment analysis of DEGs identified by the comparison between the heat treatment and control (Heat vs. CK) indicated that 73 and 694 GO terms were significantly enriched with upregulated genes (up enriched) and downregulated genes (down enriched), respectively, by the heat stress (q < 0.05; Tables S1 and S2). The top 30 up-enriched GO terms belonged to the categories of biological process and molecular function, and most of these GO terms were directly related to plants’ responses to stress and ion transport and binding (Figure 2A and Table S1). In contrast, the top 30 down-enriched GO terms belonged to the category of cellular component, and many of these GO terms were related to process and/or components of DNA packaging, chromatin/chromosome organization, cell, cell part, and organelle, etc. (Figure 2B and Table S2). 

GO enrichment analysis of DEGs identified by the comparison between the combination of heat stress and control (HF + Heat vs. CK) indicated that 86 and 813 GO terms were significantly up- and down-enriched, respectively, by the combination of heat stress and HF infestation (Tables S3 and S4). In total, 6 of these top 30 up-enriched GO terms, including response to heat stress, zinc ion transmembrane transporter activity, zinc II ion transport, raffinose alpha-galactosidase activity, zinc ion transmembrane transporter activity, and divalent inorganic cation transmembrane transporter activity, were up-enriched by heat stress (Figure 2A,C). Nevertheless, several GO terms related to plants’ response to biotic stresses were specifically found among the top 30 up-enriched GO terms due to the combination of heat stress and HF infestation, such as GO terms related to the response to biotic stimulus, chitinase activity, chitin metabolism process, and chitin catabolic process, etc. (Figure 2C). The top 30 down-enriched GO terms due to the combination of heat stress and HF infestation possess a striking similarity to those due to heat stress. Over 80% of the top 30 GO terms were found to be down-enriched by both heat stress and the combination of heat stress and HF infestation (Figure 2B,D).

Kegg pathway enrichment analysis indicated that four Kegg pathways, which include protein processing in endoplasmic reticulum, galactose metabolism, glycosphiggolipid biosypthesis-globo series, and basal transcription factors, were significantly up-enriched by both heat stress and the combination of heat stress and HF infestation (Table S5). The combination of heat stress and Hessian fly infestation also up-enriched several pathways implicated in wheat plant resistance to HF infestation, such as pathways phenylalanine metabolism [28], phenylpropanoid biosynthesis [31], and glutathione metabolism [36]. Both heat stress and the combination of heat stress and HF infestation down-enriched pathways that are central to primary metabolism, including biosynthesis of amino acids, citrate cycle, pyruvate metabolism, and carbon metabolism (Table S5). 

### 2.4. Impact of Heat Stress on Genes Upregulated by HF Infestation under Control Temperature

DEGs due to HF infestation under the control temperature represent genes associated with wheat resistance to HF infestation because ‘Molly’ wheat plants exhibit complete resistance to the infesting HF biotype *GP* at 18 °C [14]. Based on the effect of heat stress, we organized 18 HF-upregulated DEGs into 3 groups (Table S6). 

Group I included nine genes that were not affected by heat stress. Most of these genes, such as the phenylalanine ammonia lyase genes, pathogenesis-related protein 1 genes, and a chitinase IV precursor gene, were dramatically upregulated by HF infestation (Table S6). The upregulation of these genes by HF infestation but lack of regulation by heat stress suggested that the expression of these genes was uniquely associated with the resistance responses of wheat plants to HF infestation in the incompatible interaction. 

Group II included three genes upregulated by both heat stress and HF infestation (Table S6). Genes in this group included a peroxidase, a protein functioning in oxidation and defense signaling [37], and a dirigent, a protein functioning in lignan and lignin biosynthesis and cell wall fortification [38]. The upregulation of these genes by both HF infestation and heat stress suggests that they are responsive to both HF infestation and heat stress.

Group III included six genes downregulated by heat stress in the HF-infested plants (Table S6). Of these genes, the upregulation by HF infestation and downregulation by heat stress of phenylalanine ammonia lyase, aspartyl protease family protein, and carboxylesterase genes were substantial (Table S6). Because HF infestation under the normal temperature results in a wheat resistance response in ‘Molly’ wheat plants, and heat stress reduces wheat resistance efficiency [14], such results suggested that the differential regulation of these genes by HF and heat stress likely contributes to wheat resistance responses to HF infestation and the heat-induced loss of wheat resistance. 

### 2.5. Impact of Heat Stress on Genes Regulated by HF under Heat Conditions

MapMan analysis assigned 89 of 151 HF-regulated DEGs in the heat-stressed ‘Molly’ plants to 9 functional groups (Table 3). According to the mean fold increase and the number of genes involved, the most intensively upregulated group of those genes belongs to the functionary category of RNA regulation of transcription, followed by protein degradation and post-translational modification, signaling, secondary metabolism, and biotic stress (Table 3). Twenty-five of the HF-regulated genes in the heat-stressed plants were also significantly affected by heat stress. Based on the impact of heat stress, these genes were also categorized into three groups (Table S7): Group A included genes downregulated by HF but upregulated by heat stress and the combination of heat stress and HF infestation. This group included three putative small heat shock proteins of 16.9 and 17.4 Kda, respectively. The downregulation of these genes by HF infestation was mild, but the upregulation of these genes by heat stress was dramatic, with over 170-fold increases in their transcript abundance (Table S7). Given that heat stress compromises wheat resistance to HF infestation [15], the upregulation of these genes by heat stress and the combination of heat stress and HF infestation suggests that the increased expression of these heat shock protein genes may contribute to the heat-induced loss of wheat resistance to HF infestation. Group B included 11 genes upregulated by HF infestation but downregulated by heat stress (Table S7). This included seven WRKY transcription factors that play important roles in transcriptional reprogramming associated with the plant immune response [39]; an NRR repressor homolog 1 gene, a negative regulator of plant resistance [40]; a DMR6-like oxygenase gene implicated in secondary metabolism and biosynthesis of signaling molecules [41]; a dirigent protein 1-like protein that functions in biosynthesis of the cell wall component lignan [42]; and a hessian fly response gene 1 protein that has been reported to be induced in wheat plants during the incompatible interaction [27]. The downregulation of these HF-upregulated genes by heat stress may play a role in the increased wheat susceptibility to HF infestation under heat conditions. Group C included 10 genes upregulated by HF, heat stress, and the combination of heat stress and HF infestation, respectively (Table S7). The intensity of upregulation caused by the combined effect of HF and heat stress is greater than that by either HF infestation or heat stress alone. The implication of such results is two-fold: First, these genes responded to both HF infestation and heat stress; second, the more intensive upregulation of these genes in wheat plants in response to the combined effect of heat and HF infestation suggested that the effect of HF and heat stress might be additive or interactive regarding these wheat genes. This group included two 12-oxophytodienoate reductase 2 genes that may potentially be involved in OPDA degradation [43] and five lysine-specific demethylase JMJ30 isoform genes that demethylate ‘Lys-36’ (H3K36me) of histone H3 and epigenetically regulate gene expression at elevated temperatures [44]. 

## 3. Discussion

Our results provide evidence to establish the following findings: (1) Wheat genes were more responsive to HF infestation when plants were exposed to heat stress and more responsive to heat stress when plants were infested with HF (Table 1). (2) The impacts of heat stress and the combination of heat stress and HF infestation were different; however, both heat stress and the combination of heat stress and HF infestation downregulated a great number of genes in the categories of cellular component and genes involved in primary metabolic pathways (Figure 2C,D. Table S5). The striking similarity in wheat responses to heat stress and the combination of heat stress and HF infestation may partly be attributed to the not-so-dramatic effect of HF infestation caused by the small number of HF larvae feeding on wheat plants at the time of sampling [45,46,47,48,49,50,51,52,53,54,55,56]. Genome-wide transcriptome expression regarding the impact of heat stress or the impact of insect infestation on plants has been well documented in different crops [47,48,49,50]. Nonetheless, information on the transcriptome expression of plant responses to the combination of heat stress and insect pests is limited. Our findings, therefore, provide new insights regarding our understanding of the molecular basis of how host plants respond differently to insects under heat and the combined stress of insects and heat. Based on the intensity and patterns of gene regulation by HF infestation, heat stress, and/or their combination, we selected candidate genes that potentially contribute to the expression of wheat resistance to HF and/or the heat-induced loss of wheat resistance to HF infestation (Table 4).

### 3.1. Candidate Genes Potentially Contributing to Wheat Resistance to HF

We selected three candidate genes potentially contributing to wheat resistance to HF for further functional study (Table 4 and Table S6). These genes were selected because they were intensively upregulated by HF infestation in the incompatible interaction but not regulated by either heat stress or the combination of heat stress and HF infestation (Table S6). Such an expression pattern suggests that the upregulation of these genes is uniquely associated with the expression of wheat resistance during the early stage of wheat–HF infestation. These genes include a phenylalanine ammonia lyase gene (5BL_v1_408228_AA1362380) and two pathogenesis-related protein 1 genes (Table 4 and Table S6). Phenylalanine ammonia lyase is the first committed step in the phenylpropanoid pathway and is involved in the biosynthesis of polyphenol compounds such as flavonoids, phenylpropanoids, and lignin in plants [51]. Many products of phenylalanine ammonia lyase are either antibiotic/antifeedant for insects or play a role in fortifying the cell wall defense against pathogens and insects [49,51,52]; upregulation of the phenylalanine ammonia lyase gene has been implicated in the expression of host resistance and nonhost resistance to HF infestation [28,53]. Pathogenesis-related protein 1 constitutes the main family of pathogenesis-related proteins that can be induced by pathogens or salicylic acid. This type of protein possesses antifungal activities and has been found to facilitate defense responses against microbial pathogens and herbivores [54,55,56]. A previous study showed that pathogenesis-related protein 1 genes were induced by HF infestation in wheat plants in both compatible and incompatible interactions [56]. The significant upregulation of these pathogenesis-related protein 1 genes in our study by HF infestation in the incompatible interaction suggests that some specific pathogenesis-related protein 1 genes are part of the early resistance response in wheat to HF infestation. 

### 3.2. Candidate Genes Potentially Contributing to Wheat Resistance and the Heat-Induced Loss of Wheat Resistance to HF Infestation

We selected 14 candidate genes potentially contributing to both wheat resistance and the heat-induced loss of wheat resistance to HF. The first group of these genes included a phenylalanine ammonia lyase gene (1BS_v1_049914_AA0164150), an aspartyl protease family protein gene, and a carboxylesterase gene (Table 4 and Table S6). The high upregulation of these genes by HF in the incompatible interaction suggests that these genes may be necessary for the expression of wheat resistance to HF infestation. Therefore, it is logical to speculate that the high downregulation of these genes by heat stress in the HF-infested plants likely disrupts wheat resistance responses. Carboxylesterase and aspartyl protease family protein catalyze the degradation of lipids and proteins [57,58], respectively. The dramatic increase in their transcript abundance caused by HF infestation suggests the rapid degradation of lipids and proteins during the onset of wheat resistance to HF infestation. Because rapid degradation of lipids and proteins is necessary for wheat plants to mobilize resources to fortify the cell wall and produce antifeedant or toxins against HF infestation [30,31], the downregulation of the aspartyl protease family protein and the carboxylesterase genes may suggest that heat stress slows down the process of lipid and protein degradation needed for mobilizing resources in resistant wheat plants for defense purposes. The downregulation of the phenylalanine ammonia lyase gene by heat stress may suggest that heat stress hinders the process of synthesizing defensive secondary metabolites via the phenylpropanoid pathway. The second group included three small heat shock protein genes upregulated by heat stress but downregulated by HF infestation in the heat-stressed plants (Table 4 and Table S7). One of these heat shock proteins (3as_tgacv1_210681_aa0677000) is 96% identical in its nucleotide sequence to the wheat HF susceptibility gene *Mds-1* (GenBank: JN162442.1) [12] and has been mapped to the same chromosome arm 3AS. The other two small heat shock protein genes (4dl_tgacv1_342934_aa 1125640) and 5BL (5bl_tgacv1_404396_aa1298380) were mapped in the chromosome 4DL (Table 4 and Table S7). Because the pattern of gene regulation of these small heat shock proteins was similar to that of *Mds-1* [12], we speculate that upregulation of these small heat shock protein genes may also confer susceptibility in wheat to HF infestation. The third group includes eight genes upregulated by HF infestation in the heat-stressed plants but downregulated by heat stress (Table 4 and Table S7). The upregulation of these genes by HF under heat conditions suggests that they may be resistant responsive genes, and the downregulation of these genes by heat stress suggests that heat stress suppresses the expression of these resistance responsive genes, causing susceptibility in ‘Molly’ wheat plants to HF infestation. These genes include four WRKY transcription factor 76/76-like genes, an NRR homolog 1 gene, a DMR6-like oxygenase 1 gene, a dirigent protein 21-like gene, and a Hessian fly response gene 1. WRKY transcription factors play a significant role in transcriptional reprogramming in relation to plant immune responses and are key regulators of secondary metabolites, including phenylpropanoids, indole alkaloids, and terpenoids [59]. The upregulation of many WRKY transcription factor was found in *Brachypodium distachyon*, a wheat-related species that exhibits nonhost resistance to Hessian fly [53]. The downregulation of these WRKY transcription factors by heat stress in wheat plants suggests that heat stress may suppress the synthesis of defensive secondary metabolites via suppression of the expression of WRKY transcription factor genes (Table S7). The *NRR* gene is a negative regulator of plant resistance in rice. When constituently expressed, the *NRR* gene affects basal resistance, age-related resistance, and Xa21-mediated resistance in rice, causing enhanced susceptibility to the pathogen *Xanthomonasoryzae* pv. *Oryzae* [40]. Nevertheless, the role of the *NRR* gene in plant resistance to insects remains largely unknown. DMR6-like oxygenase belongs to the superfamily of 2-oxoglutarate Fe (II)-dependent oxygenases, which are implicated in secondary metabolism and the biosynthesis of signaling molecules, e.g., the biosynthesis of flavonoids, gibberellins, and alkaloids [41]. Dirigent proteins impart stereoselectivity on the phenoxy radical-coupling reaction, yielding optically active lignans from two molecules of coniferyl alcohol in the biosynthesis of lignans, flavonolignans, and alkaloids; thus, it plays a central role in plant secondary metabolism and cell wall fortification [60]. The downregulation of DMR6 and dirigent protein genes suggests that heat stress may delay the process of producing defensive secondary metabolites and weaken cell wall fortification. Hessian fly responsive gene 1 was induced in wheat plants during the incompatible interaction, and its protein possesses lectin-like domains and insecticide activity [61]. The downregulation of Hessian fly responsive gene 1 by heat stress in our study may suggest weaker antibiotic resistance in heat-stressed wheat plants to HF infestation. 

To summarize, our results suggest that wheat genes in plants exposed to heat stress are more sensitive to HF infestation, and that wheat genes in HF-infested plants are more responsive to heat stress. Via analysis of the impact of heat stress on genes affected by HF under normal and heat-stressed conditions, we selected 17 candidate genes that may contribute to wheat resistance to HF infestation and/or the heat-induced loss of wheat resistance during the early stage of wheat–Hessian fly interaction. Further studies are necessary to establish a direct connection between the expression of these candidate genes and the expression of wheat resistance and susceptibility to HF under normal and heat-stressed conditions. 

## 4. Materials and Methods

### 4.1. Plant Preparation and Infestation

The wheat cultivar ‘Molly’ and a Hessian fly (HF) population named ‘White eyes’ were used in this study. ‘Molly’ possesses a HF resistance gene *H13* [5]. The HF population consisted chiefly of the avirulent biotype *GP*. ‘Molly’ exhibited complete resistance to the ‘White eyes’ population at room or lower temperatures [15]. To prepare the plants, 20 germinated ‘Molly’ seeds were planted in each pot with a 10-cm diameter filled with potting mix (Scotts Miracle-Gro Company, Marysville, OH, USA). The plants were grown in a growth chamber set at 18 °C and a 14:10 (day: night) photoperiod until most plants reached the 1.5 leaf stage. Wheat seedlings were thinned so that each pot contained 15 seedlings of a similar growth stage. To infest the plants, eight newly emerged female adult HFs and two male adult HFs were released onto plants in each pot confined within a cage. The female adult HFs laid eggs on the leaves of wheat seedlings. The eggs developed into larvae, which crawled down to the base of the plants and attacked the plants there. To determine the time when larvae began to attack the plants, some infested plants (checking plants) were dissected and observed hourly under a dissection microscope beginning at 96 h following the release of the adult HFs. The time when HF larvae were first seen at the base of a plant was taken as the time for initial HF larval attack (0-time point). 

### 4.2. Treatment and Experimental Design 

Four treatments were applied in this experiment: (1) control plants grown under 18 °C (CK), (2) plants exposed to heat stress of 35 °C for 6 h (Heat), (3) plants infested with HF under the control temperature (HF), and (4) plants infested with HF and exposed to heat stress of 35 °C for 6 h (HF + Heat). The experiment was conducted in two Percival growth chambers (Perry, IA 50220) following a complete randomized block design (CRBD) with three biological replicates. All plants grew at 18 °C except during the time of the heat treatment.

### 4.3. Application of Heat Treatment and Sampling

Heat stress was applied by placing plants in a growth chamber set at 35 °C. The heat treatment began when the HF-infested plants reached the 0-time point of the wheat–HF interaction. Sampling began right after the completion of the heat treatment. For plants infested with HF, samples were only harvested from HF feeding site tissues in plants containing 2–5 HF feeding larvae. To collect samples, each wheat seedling was cut from its base. The second leaf sheath was carefully peeled off and rinsed in water to remove larvae from infested plants and then dried with a piece of paper towel to remove excessive water. A 10-mm section of each second leaf sheath was collected into a 1.5-mL Eppendorf tube filled with RNAlater (Thermal Fisher Scientific, Waltham, MA, USA). Samples were stored at −20 °C before shipping to a commercial sequencing facility for RNA extraction, library construction, and sequencing.

### 4.4. RNA Extraction, Library Construction, and Sequencing

RNA was extracted using Trizol Reagent (ThermoFisher Scientific, Waltham, MA, USA) following the manufacturer’s instructions. Libraries were generated using a NEBNext^®^ Ultra™ RNA Library Prep Kit for Illumina^®^ (New England Biolabs, Ipswich, MA, USA) following the manufacturer’s recommendations. Briefly, mRNA was purified from total RNA using poly-T oligo-attached magnetic beads. Fragmentation was carried out using divalent cations under an elevated temperature in NEBNext First Strand Synthesis Reaction Buffer 5X. First-strand cDNA was synthesized using random hexamer primer and M-MuLV Reverse Transcriptase (RNase H-). Second-strand cDNA synthesis was performed using DNA Polymerase I and RNase H followed by a round of purification, terminal repair, A-tailing, ligation of sequencing adapters, size selection, and PCR enrichment. The library concentration was first quantified using a Qubit 2.0 fluorometer (Life Technologies, Carlsbad, CA, USA) and then diluted to 1 ng/μL before checking the insert size on an Agilent 2100 system (Agilent Technologies, Santa Clara, CA, USA). Libraries were sequenced using an Illumina HiSeq-PE150 sequencing platform. The RNA-Seq data were deposited in the BioProject with the accession number PRJNA589693 (https://www.ncbi.nlm.nih.gov/bioproject/ (accessed on 14 November 2019)).

### 4.5. Quality Control, Sequencing Alignment, and Quantification of the Transcript Abundance

Raw reads in fastq format were filtered to remove the reads containing adapters or reads of low quality to produce clean reads. Paired-end clean reads were then mapped to the *Triticum aestivum* genome sequence (ftp://ftp.ensemblgenomes.org/pub/plants/re-lease-39/gtf/triticum_aestivum/ (accessed on 14 November 2019)) using HISTAT [62]. Only uniquely mapped reads were used for further read counting per gene, normalization of read counts, and gene expression analyses. HTSeq v0.6.1 was used to count the reads numbers mapped to each gene [63]. FPKM, the number of fragments per kilobase of transcript sequence per millions base pairs sequenced of each gene, was calculated based on the length of the gene and reads count mapped to the gene [64]. Fold changes in the transcript abundance of a gene between a treatment and the control or two different treatments were expressed as the normalized read count of the gene.

### 4.6. Differential Expression Analysis

Differential expression analyses were performed on normalized read counts using DESeq R package [65]. The resulting *p*-values were adjusted using the Benjamini and Hochberg’s approach for controlling the false discovery rate [66]. Genes with an adjusted *p*-value (q value) < 0.05 were considered differentially expressed. 

### 4.7. GO and KEGG Enrichment Analysis 

Gene Ontology (GO, http://www.geneontology.org (accessed on 14 November 2019)) enrichment analysis was implemented using GOseq R package, in which gene length bias was corrected [67]. GO terms with q value < 0.05 were considered significantly enriched by differentially expressed genes (DEGs). KEGG (Kyoto Encyclopedia of Genes and Genomes) pathways were retrieved (http://www.genome.jp/kegg/ (accessed on 14 November 2019)), and KOBAS software was used to test the statistical significance of the enrichment of DEGs in KEGG pathways. 

### 4.8. Blast to Annotate Transcripts

Sequences of transcripts were searched for against the GenBank non-redundant nucleotide database (nr/nt) using BLASTN to identify homologous hits via Blast2Go (https://www.blast2go.com/ (accessed on 14 November 2019)) for each transcript. Only the best hit with an E-value no larger than 1E−30 was selected.

### 4.9. MapMan Analysis 

MapMan was specifically designed to cover plant-specific pathways and processes (https://mapman.gabipd.org/mapman (accessed on 14 November 2019)). MapMan pathway analyses were performed on the Log2 fold change of differentially expressed genes between the treatments [68]. A custom specific mapping file for the MapMan based on the wheat sequencing output was created using the Mercator pipeline [69] (http://mapman.gabipd.org/web/guest/mercator (accessed on 14 November 2019)) in which TAIR, PPAP, KOG, CDD, ORYZA, and IPR, BLAST CUTOFF of 80, and ANNOTATE options were selected as parameters for the transcript annotation to obtain the hierarchical BIN (functional) categories. The functions or putative functions of the sequences were assigned by the BLAST-based search function of Mercator based on the reference proteins and functional domains of the sequences.

### 4.10. RT qPCR Analysis

Including the nontreated control, the second leaf sheath of wheat plants at the 1.5 leaf stage was harvested after the heat (35 °C for 6 h), HF infestation at normal temperature (18 °C), and heat plus HF infestation treatments, respectively, and immediately stored in a 1.5-mL Eppendorf tube filled with RNAlater (Thermal Fisher Scientific, Waltham, MA, USA). These samples were used to extract total RNA using a Qiagen RNease Plant Mini kit (Germantown, MD, USA). The RT-qPCR primers of DGEs were designed by Gene Runner software [70] using customer preferred parameters. Two pairs of primers from heat shock protein 2 (HSP2), jasmonate ZIM domain (JAZ), and phenylalanine ammonia lyase (PAL) genes, respectively, were selected from different exon combinations as the target genes, and two pairs of primers from the wheat actin gene were designed as the reference gene (Table 2). The cDNA was synthesized using the ThermalFisher’s SuperScript™ IV One-Step RT-PCR System (Waltham, MA, USA) according to the manufacturer’s protocol. RT-qPCR) was conditioned with 5 μL of PowerUp™ SYBR™ Green Master Mix (ThermalFisher), 0.5 μL of each primer (0.5 μM), and 1 μL of template, and ddH_2_O was added until a 10 μL total volume was reached. Three technical replicates were required for each sample. RT qPCR was performed under a standard two steps of the PCR condition using the Applied Biosystems 7500 Sequence Detection System (ABI, MA, USA). The relative expression level was quantified using the 2^−ΔΔCt^ method [71].

## Figures and Tables

**Table 1:** Number of DEGS due to heat stress (Heat vs. CK), heat stress in the HF-infested plants (HF + Heat vs. HF), HF infestation (HF vs. CK), HF infestation in the heat-stressed plants (HF + Heat vs. Heat), and the combined effect of heat stress and HF infestation (HF + Heat vs. CK).

	Heat vs. CK	HF + Heat vs. HF	HF vs. CK	HF + Heat vs. Heat	HF + Heat vs. CK
**Total DEGs**	4197	11,675	21	155	7164
**Upregulated**	1892	5238	18	151	3793
**Downregulated DEGS**	2305	6437	3	4	3371

**Table 2:** Genes and oligonucleotide sequences of primer pairs used for real-time qPCR.

Primer Name	Gene ID	Gene Name	Oligo Sequence
HSP2_F1	TRIAE_CS42_3AS_TGACv1_213275_AA0706340	HSP2	TGAGGTTCTTCGACACGCTGGCCCT
HSP2_R1	TRIAE_CS42_3AS_TGACv1_213275_AA0706340	HSP2	TCCTCCTCCACCTCCACCTTGACCTCC
HSP2_F2	TRIAE_CS42_3AS_TGACv1_213275_AA0706340	HSP2	CATCTTCTGCACCACGGCGTCCAGC
HSP2_R2	TRIAE_CS42_3AS_TGACv1_213275_AA0706340	HSP2	AGCACCTTGCCGTCCTCCTCCACCT
PAL1_F1	TRIAE_CS42_1BS_TGACv1_049914_AA0164150	PAL1	GCGTCAAGGAGAGCAGCGACTGGGTCA
PAL1_R1	TRIAE_CS42_1BS_TGACv1_049914_AA0164150	PAL1	AGCGCCGCCCTCCTTGGTCCTCC
PAL1_F2	TRIAE_CS42_1BS_TGACv1_049914_AA0164150	PAL1	GGAGGACCAAGGAGGGCGGCGCT
PAL1_R2	TRIAE_CS42_1BS_TGACv1_049914_AA0164150	PAL1	CGGCAGGCATGGTGTCACGTTGGC
JAZ_F1	TRIAE_CS42_5AL_TGACv1_379331_AA1256080	JAZ	AGCAGCAGCCTCGTTCAGCAGAGCCCT
JAZ_R1	TRIAE_CS42_5AL_TGACv1_379331_AA1256080	JAZ	GCCCAGCCCGAGCCAGGAGTTGTCA
JAZ_F2	TRIAE_CS42_5AL_TGACv1_379331_AA1256080	JAZ	TCCCCCTCGAGAAATCTAGTGTTGGCCAG
JAZ_R2	TRIAE_CS42_5AL_TGACv1_379331_AA1256080	JAZ	AGGGCTCTGCTGAACGAGGCTGCTGCT
Wh_Act_F1	Traes_1AS_A8AD3BE99.2	Actin7	TACAATGAGCTCCGTGTGGCACCTGAGG
Wh_Act_F2	Traes_1AS_A8AD3BE99.3	Actin7	CGGTATCGTAAGCAACTGGGATGACATGGAG
Wh_Act_R1	Traes_1AS_A8AD3BE99.4	Actin7	CTCGGTGAGGATCTTCATGAGGGAGTCCG
Wh_Act_R2	Traes_1AS_A8AD3BE99.5	Actin7	ACAATTTCCCGCTCGGCTGAGGTTGTG

**Table 3:** Number, mean value, maximum, and minimum fold change of the transcript abundance of major functional groups of annotated DEGs affected by HF infestation in heat-stressed plants.

Function Group	Number	Mean	Maximum	Minimum
Secondary metabolism	10	14.9	45.9	3.0
Hormone metabolism	5	7.0	14.4	2.6
Stress: Biotic	15	10.8	20.6	2.6
Stress: Abiotic heat shock protein	3	0.24	0.16	0.33
RNA regulation of transcription	20	41.5	246.4	2.3
Protein: post-translational modification/degradation	13	34.9	150.1	3.0
Signaling	11	22.4	67.0	2.9
Development	7	8.1	12.1	5.2
Transport	5	9.0	15.4	3.6

**Table 4:** Selected candidate genes potentially contributing to wheat resistance to HF infestation and/or the heat-induced loss of wheat resistance.

Gene Id	Gene Description
**Candidate genes potentially contribute to wheat resistance to HF infestation**	
5BL_v1_408228_AA1362380	Phenylalanine ammonia lyase
2AL_v1_109444_AA0328580	Pathogenesis-related protein 1
2AL_v1_094607_AA0300380	Pathogenesis-related protein 1
**Candidate genes potentially contribute to wheat resistance to HF infestation and the heat-induced loss of wheat resistance**	
1BS_v1_049914_AA0164150	Phenylalanine ammonia lyase
7DL_v1_603712_AA1987770	Aspartyl protease family protein
7DS_v1_622332_AA2037800	Carboxylesterase
3as_tgacv1_210681_aa0677000	16.9 kDa class I heat shock protein 1-like
4dl_tgacv1_342934_aa1125640	17.9 kDa class I heat shock protein
5bl_tgacv1_404396_aa1298380	16.9 kDa class I heat shock protein 1-like
5al_tgacv1_373966_aa1185670	WRKY transcription factor WRKY76-like
5bl_tgacv1_407645_aa1358580	WRKY transcription factor WRKY76
5dl_tgacv1_436840_aa1463530	WRKY transcription factor WRKY76
5dl_tgacv1_432937_aa1395210	WRKY transcription factor WRKY76
1bl_tgacv1_034437_aa0144510	NRR repressor homolog 1
2al_tgacv1_093272_aa0276390	DMR6-LIKE OXYGENASE 1 (flavonoids metabolism)
2ds_tgacv1_177854_aa0586060	Dirigent protein 21-like
7ds_tgacv1_621992_aa2030750	Hessian fly response gene 1 protein

## Data Availability

Not applicable.

## Supplementary Materials

The following supporting information can be downloaded at: https://www.mdpi.com/article/10.3390/plants11111402/s1, Table S1: Significantly enriched GO terms with upregualted DEGs due to heat stress (Heat vs. CK comparison); Table S2: Significantly enriched GO terms with downregulated DEGs due to heat stress (Heat vs. CK comparison); Table S3:Significantly enriched GO terms with upregualted DEGs due to the combination of heat stress and HF infestation (HF+Heat vs. CK comparison); Table S4:Significantly enriched GO terms with downregualted DEGs due to the combination of heat stress and HF infestation (HF+Heat vs. CK comparison); Table S5:Kegg pathways significantly enriched by heat stress and the combination of heat stress and HF infestation, respectively (q < 0.05).; Table S6:DEGs upregulated by HF infestation under normal temperature and fold changes in transcript abundance of these genes due to HF infestation (HF vs. CK), heat stress (Heat vs. CK), the combination of heat stress and HF infestation (HF+Heat vs. CK), and heat stress in the HF infested plants (HF+Heat vs. HF); Table S7:Fold change in transcript abundance caused by heat stress (Heat vs. CK) and the combination of heat stress and HF infestation (HF+Heat vs. CK) on genes that are regulated by HF infestation in heat stressed plants (HF+Heat vs. Heat).

## Abbreviations

HF	Hessian fly
DEGs	Differentially expressed genes
GO	Gene ontology
KEGG	Kyoto Encyclopedia of Genes and Genomes
