# Supplementary material for: Impact of Heat Stress on Expression of Wheat Genes Responsive to Hessian Fly Infestation
Source: Plants (Basel). 2022 May 25;11(11):1402. doi: 10.3390/plants11111402 (PMC9183101; doi:10.3390/plants11111402)
Supplement: Supplementary file 1 [file plants-11-01402-s001.zip › Table S5.pdf]

Table S5. Kegg pathways significantly enriched by heat stress and the combination of heat stress and HF infestation, respectively (q < 0.05).

Term	ID	Input number	Background number	q-value
**Pathways up enriched by heat stress (Heat vs CK)**				
Protein processing in endoplasmic reticulum	osa04141	70	203	2.77E-22
Galactose metabolism	osa00052	20	54	1.47E-06
Spliceosome	osa03040	34	196	0.000184
Glycosphingolipid biosynthesis - globo series	osa00603	6	8	0.003251
Basal transcription factors	osa03022	10	41	0.026583
**Pathways down enriched by heat stress (Heat vs CK)**				
Carbon metabolism	osa01200	73	225	0.038981
Citrate cycle (TCA cycle)	osa00020	25	50	0.017641
Pyruvate metabolism	osa00620	33	75	0.017641
Fatty acid metabolism	osa01212	33	72	0.01433
Fatty acid biosynthesis	osa00061	25	40	0.0025
Biosynthesis of secondary metabolites	osa01110	213	779	0.037688
Biosynthesis of amino acids	osa01230	84	207	0.000409
Cysteine and methionine metabolism	osa00270	43	85	0.001003
Biotin metabolism	osa00780	17	20	0.0025
**Pathways up enriched by the combination of heat stress and HF infestation (HF+Heat vs CK)**				
Protein processing in endoplasmic reticulum	osa04141	75	203	7.12E-12
Galactose metabolism	osa00052	22	54	0.00080706
Glutathione metabolism	osa00480	25	80	0.00528206
Phenylpropanoid biosynthesis	osa00940	32	125	0.01135889
Phenylalanine metabolism	osa00360	26	99	0.02207683
Stilbenoid, diarylheptanoid and gingerol biosynthesis	osa00945	9	18	0.03074527
Basal transcription factors	osa03022	14	41	0.03157745
Glycosphingolipid biosynthesis - globo series	osa00603	6	8	0.03218391
Starch and sucrose metabolism	osa00500	32	145	0.0397822
**Pathways down enriched by the combination of heat stress and HF infestation (HF+Heat vs CK)**				
Pyruvate metabolism	osa00620	57	75	0.00011171
Fatty acid metabolism	osa01212	53	72	0.00018849
Biosynthesis of amino acids	osa01230	112	207	0.00018849
Carbon metabolism	osa01200	118	225	0.00020306
Citrate cycle (TCA cycle)	osa00020	41	50	0.00020306
Glycolysis / Gluconeogenesis	osa00010	71	119	0.00050205
DNA replication	osa03030	34	54	0.02583402
Cysteine and methionine metabolism	osa00270	47	85	0.02611459
