# Supplementary material for: Impact of Heat Stress on Expression of Wheat Genes Responsive to Hessian Fly Infestation
Source: Plants (Basel). 2022 May 25;11(11):1402. doi: 10.3390/plants11111402 (PMC9183101; doi:10.3390/plants11111402)
Supplement: Supplementary file 1 [file plants-11-01402-s001.zip › Table S6.pdf]

Table S6. DEGs upregulated by HF infestation under normal temperature and fold changes in transcript abundance of these genes due to HF infestation (HF vs CK), heat stress (Heat vs CK), the combination of heat stress and HF infestation (HF+Heat vs CK), and heat stress in the HF infested plants (HF+Heat vs HF).

Gene Id[a]	Gene description	HF vs CK	Heat vs CK	HF+Heat vs CK	HF+Heat vs HF
**I. Genes uniquely upregulated by HF infestation but not responsive to heat stress**					
7AL_v1_556139_AA1756710	Translation initiation factor IF-2	Inf (18.64/0)[b]	ns[c]	15.6	ns
5BL_v1_408228_AA1362380	Phenylalanine ammonia lyase	Inf (14.0/0)	ns	ns	ns
1DS_v1_080107_AA0239340	Phenylalanine ammonia lyase	Inf (36.2/0)	ns	Inf (19/0)	ns
2AL_v1_109444_AA0328580	Pathogenesis-related protein 1	Inf (14.8/0)	ns	ns	ns
2AL_v1_094607_AA0300380	Pathogenesis-related protein 1	Inf (22.4/0)	ns	ns	ns
5AL_v1_379331_AA1256080	Jasmonate ZIM domain-containing protein (JAZ)	5.9	ns	ns	ns
4BL_v1_320705_AA1046770	Xyloglucan hydrolase protein 31	6.0	ns	ns	ns
2AL_v1_094147_AA0293320	Chitinase IV precursor	49.9	ns	24.5	ns
1AL_v1_003726_AA0051220	CASP-like protein	Inf (16.9/0)	ns	Inf (8/0)	ns
**II. Genes upregulated by both HF infestation and heat stress**					
2BS_v1_146256_AA0460570	Peroxidase	Inf (48.3/0)	In (15.7 /0)	ns	ns
2DS_v1_177965_AA0588260	Dirigent protein	19.03	24.9	37.1	ns
3B_T1_221042_AA0728000	No hit	11.54	5.9	12.7	ns
**III. Genes up-regulated by HF infestation but down-regulated by heat stress in the HF infested plants**					
1BS_v1_049914_AA0164150	Phenylalanine ammonia lyase	Inf (41.9/0)	ns	ns	0.006
1DL_v1_061484_AA0196620	RmlC-like cupins superfamily protein	5.8	ns	ns	0.281
3AS_v1_213275_AA0706340	17.2 kDa heat shock protein	8.4	ns	ns	0.349
7DL_v1_603712_AA1987770	Aspartyl protease family protein	18.0	ns	ns	0.056
7DS_v1_622332_AA2037800	Carboxylesterase	Inf (28.9/0)	ns	ns	0.049
1DL_v1_061987_AA0206950	Glucan endo-1,3-beta-glucosidase	43.3	ns	16.0	0.412

[a]: the prefix of gene ID triae_cs42_4dl_tgacv1is the same for all genes and omitted.

[b]: Inf: fold changes are infinite because of the expression of the gene was not detected in the control plants (CK); expressed as the normalized read count/0.

[c]: ns: Not significant statistically at $q \leq 0.05$.
