# Supplementary material for: Impact of Heat Stress on Expression of Wheat Genes Responsive to Hessian Fly Infestation
Source: Plants (Basel). 2022 May 25;11(11):1402. doi: 10.3390/plants11111402 (PMC9183101; doi:10.3390/plants11111402)
Supplement: Supplementary file 1 [file plants-11-01402-s001.zip › Table S7.pdf]

Table S7. Fold change in transcript abundance caused by heat stress (Heat vs CK) and the combination of heat stress and HF infestation (HF+Heat vs CK) on genes that are regulated by HF infestation in heat stressed plants (HF+Heat vs Heat).

Gene ID[a]	Gene description	HF+Heat vs Heat	Heat vs CK	HF+Heat vs CK
**Group A: Genes downregulated by HF but upregulated by heat stress and the combination of heat stress and HF infestation**				
3as_tgacv1_210681_aa0677000	16.9 kDa class I heat shock protein 1-like	0.214	Inf (45.9/0)[b]	Inf (10/0)
4dl_tgacv1_342934_aa1125640	17.9 kDa class I heat shock protein	0.164	171.4	28.1
5bl_tgacv1_404396_aa1298380	16.9 kDa class I heat shock protein 1-like	0.325	186.7	61.3
7as_tgacv1_571537_aa1848440	ATP-dependent zinc metalloprotease FTSH 6, chloroplastic	0.255	174.2	45.0
**Group B: Genes upregulated by HF but downregulated by heat stress under control temperature**				
5al_tgacv1_373966_aa1185670	WRKY transcription factor WRKY76-like	246.4	0.023	5.8
5bl_tgacv1_407645_aa1358580	WRKY transcription factor WRKY76	155.1	0.037	5.8
5dl_tgacv1_436840_aa1463530	WRKY transcription factor WRKY76	104.4	0.086	8.9
5dl_tgacv1_432937_aa1395210	WRKY transcription factor WRKY76	137.7	0.015	ns[c]
2dl_tgacv1_158819_aa0526620	putative WRKY transcription factor 70	14.9	0.152	ns
5al_tgacv1_376168_aa1233160	putative WRKY transcription factor 58	20.7	0.098	ns
1bl_tgacv1_034437_aa0144510	NRR repressor homolog 1	23.8	0.063	ns
2al_tgacv1_093272_aa0276390	DMR6-LIKE OXYGENASE 1 (flavonoids metabolism)	30.2	0.051	ns
2ds_tgacv1_177854_aa0586060	dirigent protein 21-like	14.0	0.172	ns
7ds_tgacv1_621992_aa2030750	hessian fly response gene 1 protein	23.4	0.093	ns
2ds_tgacv1_177565_aa0580190	probable 5'-adenylylsulfate reductase 1, chloroplastic	2.9	0.151	0.436
**Group C: Genes upregulated by HF, heat stress, and the combination of heat stress and HF infestation, respectively**				
6bl_tgacv1_502794_aa1626450	putative 12-oxophytodienoate reductase 11	3.7	55.3	204.2
6dl_tgacv1_527350_aa1702600	putative 12-oxophytodienoate reductase 11	4.3	24.3	104.7
4as_tgacv1_307326_aa1019500	UDP-glycosyltransferase 83A1	4.3	18.9	81.6
4dl_tgacv1_343563_aa1136610	UDP-glycosyltransferase 83A1	3.5	25.9	90.3
5al_tgacv1_374195_aa1193180	lysine-specific demethylase JMJ30 isoform X1	3.4	3.3	11.5
5bl_tgacv1_404509_aa1302530	lysine-specific demethylase JMJ30 isoform X2	2.8	3.2	9.0
5dl_tgacv1_435238_aa1447590	lysine-specific demethylase JMJ30 isoform X1	3.0	4.1	12.3
7bs_tgacv1_592357_aa1936350	lysine-specific demethylase JMJ30-like isoform X2	3.0	7.9	24.2
7ds_tgacv1_624042_aa2058470	lysine-specific demethylase JMJ30-like isoform X1	2.6	11.1	29.4
4bs_tgacv1_329179_aa1098640	probable E3 ubiquitin-protein ligase ARI1	3.0	11.0	33.2

[a]: the prefix of gene ID triae_cs42_4dl_tgacv1is the same for all genes and omitted.

[b]: Inf: fold changes are infinite because of the expression of the gene was not detected in the control plants (CK); expressed as the normalized read counts/0.

[c]: ns: Not significant statistically at q ≤ 0.05.
